# Supplementary material for: A Prospective Study of Longitudinal Risks of Cognitive Deficit for People Undergoing Glioblastoma Surgery Using a Tablet Computer Cognition Testing Battery: Towards Personalized Understanding of Risks to Cognitive Function
Source: J Pers Med. 2023 Jan 31;13(2):278. doi: 10.3390/jpm13020278 (PMC9967594; doi:10.3390/jpm13020278)
Supplement: Supplementary file 1 [file jpm-13-00278-s001.zip › Supplementary_Figures.pptx]

## Slide 1
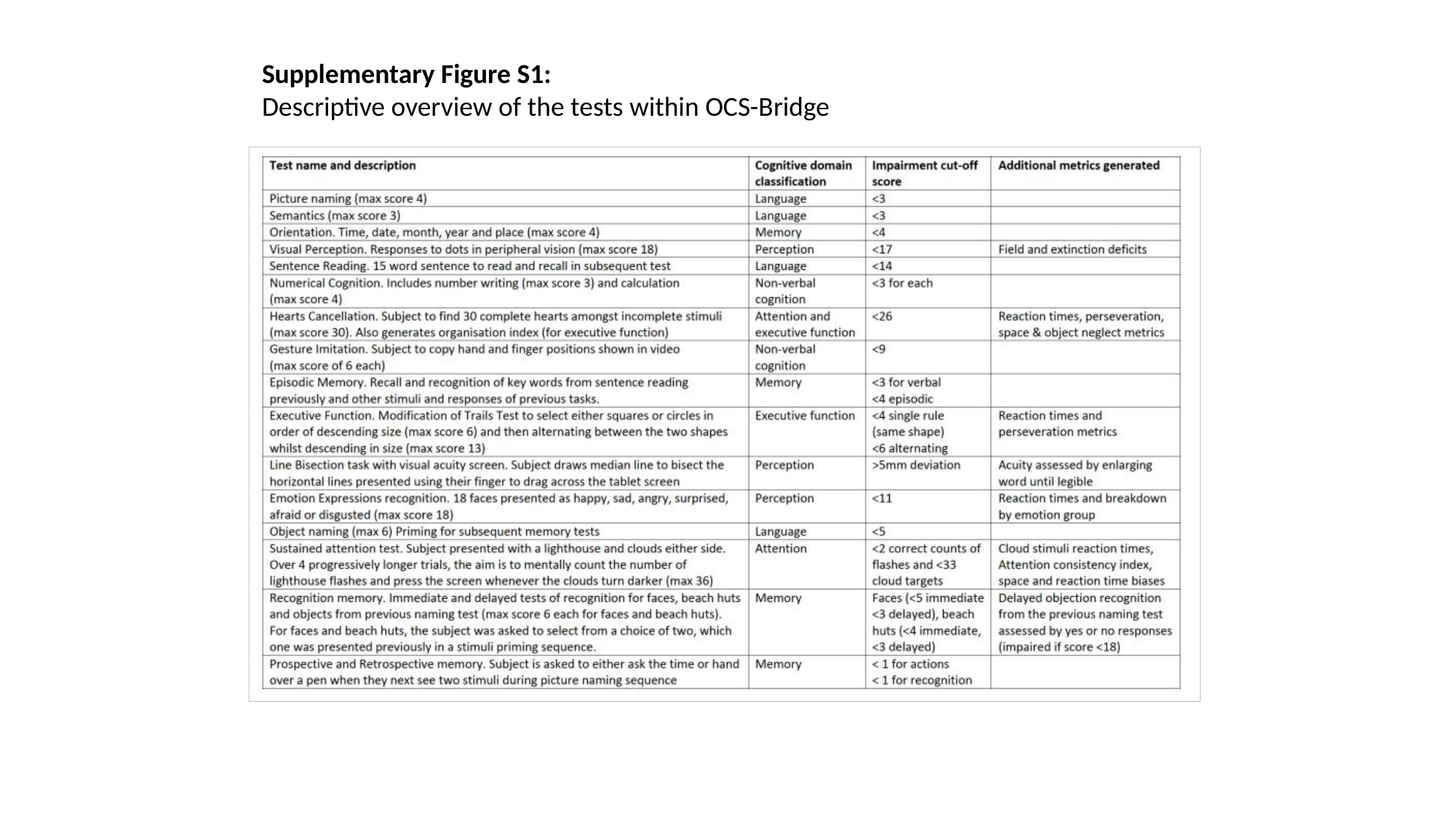

Supplementary Figure S1:
Descriptive overview of the tests within OCS-Bridge

## Slide 2
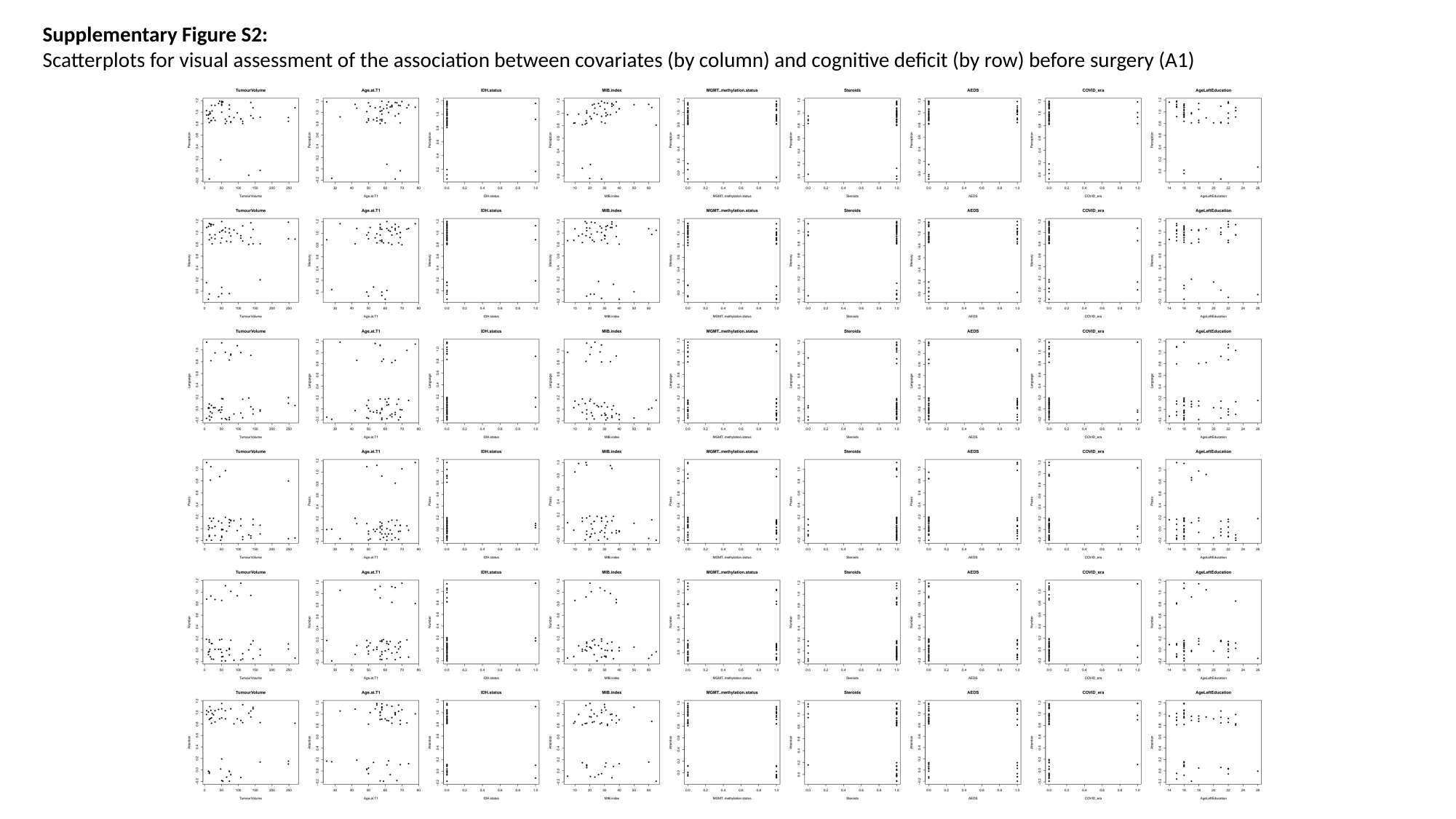

Supplementary Figure S2:
Scatterplots for visual assessment of the association between covariates (by column) and cognitive deficit (by row) before surgery (A1)

## Slide 3
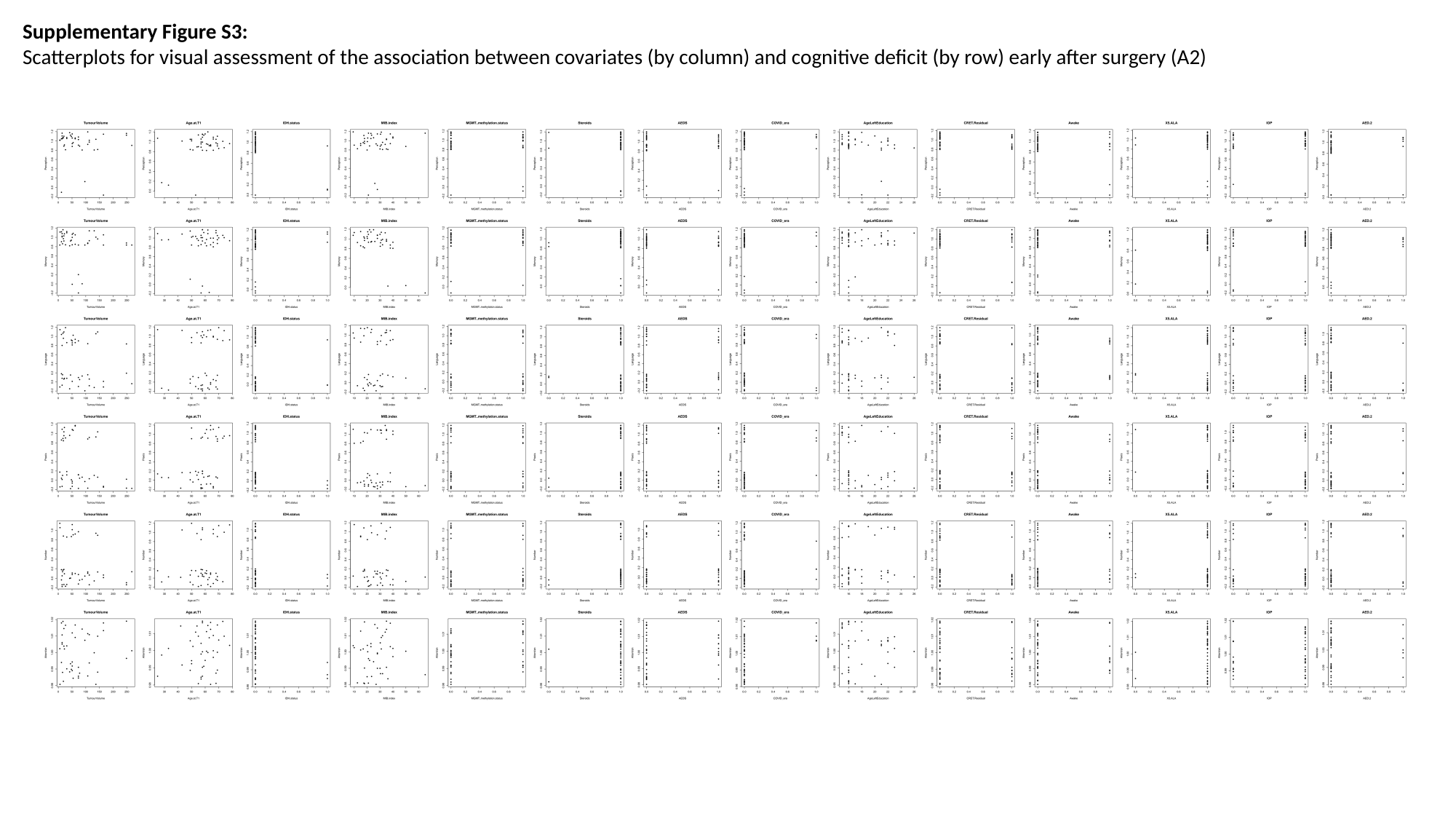

Supplementary Figure S3:
Scatterplots for visual assessment of the association between covariates (by column) and cognitive deficit (by row) early after surgery (A2)

## Slide 4
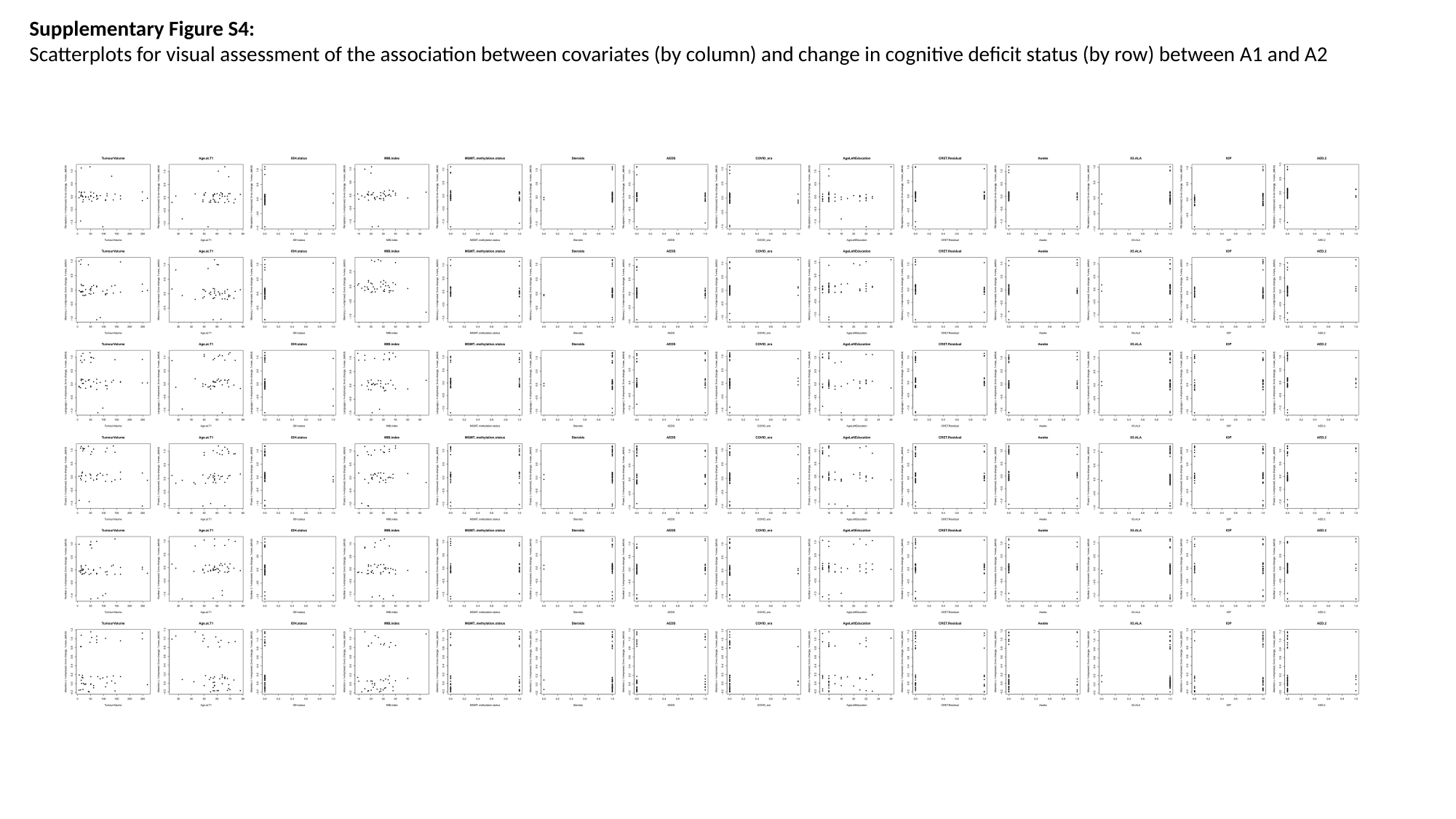

Supplementary Figure S4:
Scatterplots for visual assessment of the association between covariates (by column) and change in cognitive deficit status (by row) between A1 and A2

## Slide 5
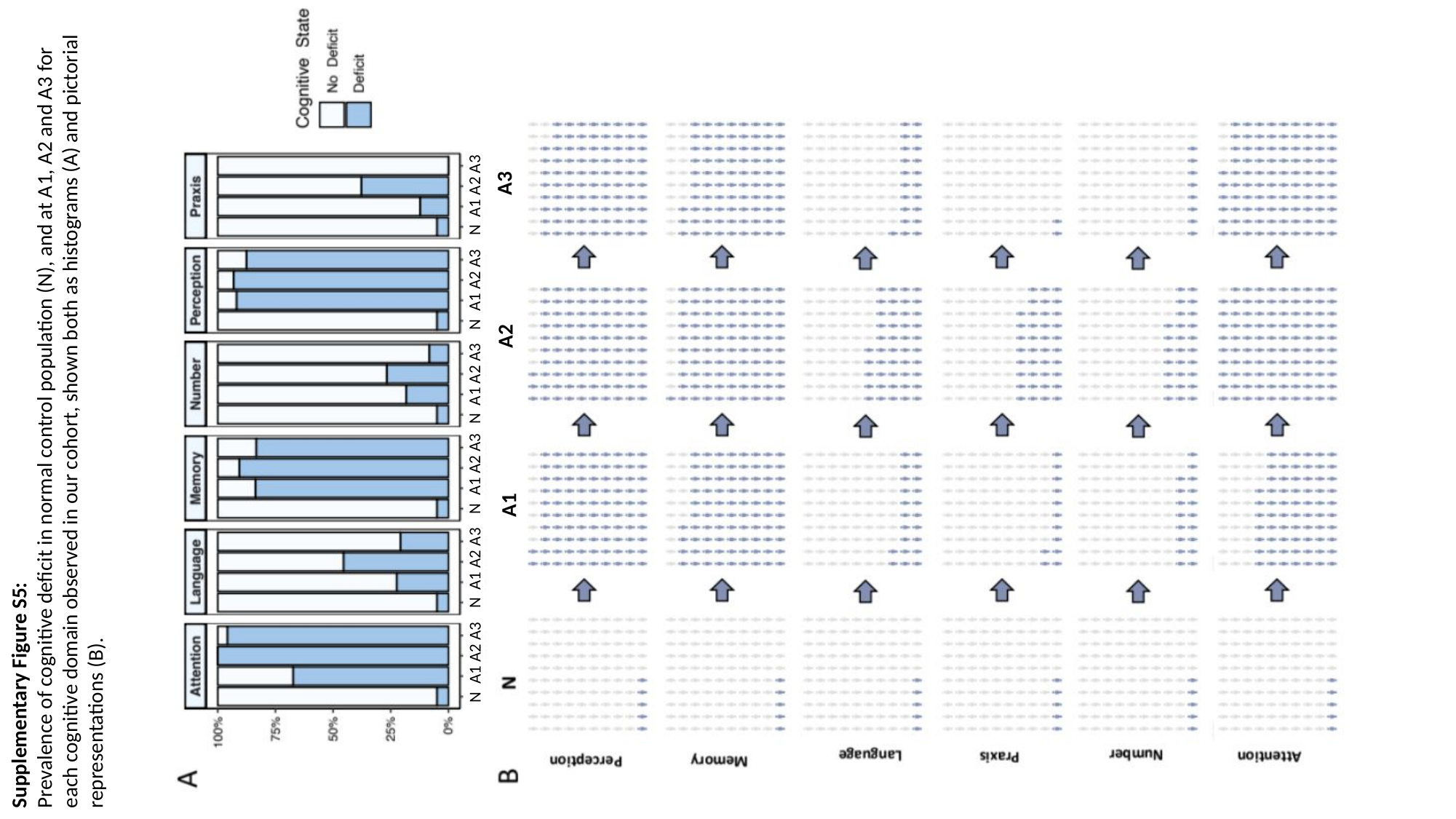

A3
N A1 A2 A3
N A1 A2 A3
A2
Supplementary Figure S5:
Prevalence of cognitive deficit in normal control population (N), and at A1, A2 and A3 for each cognitive domain observed in our cohort, shown both as histograms (A) and pictorial representations (B).
N A1 A2 A3
N A1 A2 A3
A1
N A1 A2 A3
N A1 A2 A3

## Slide 6
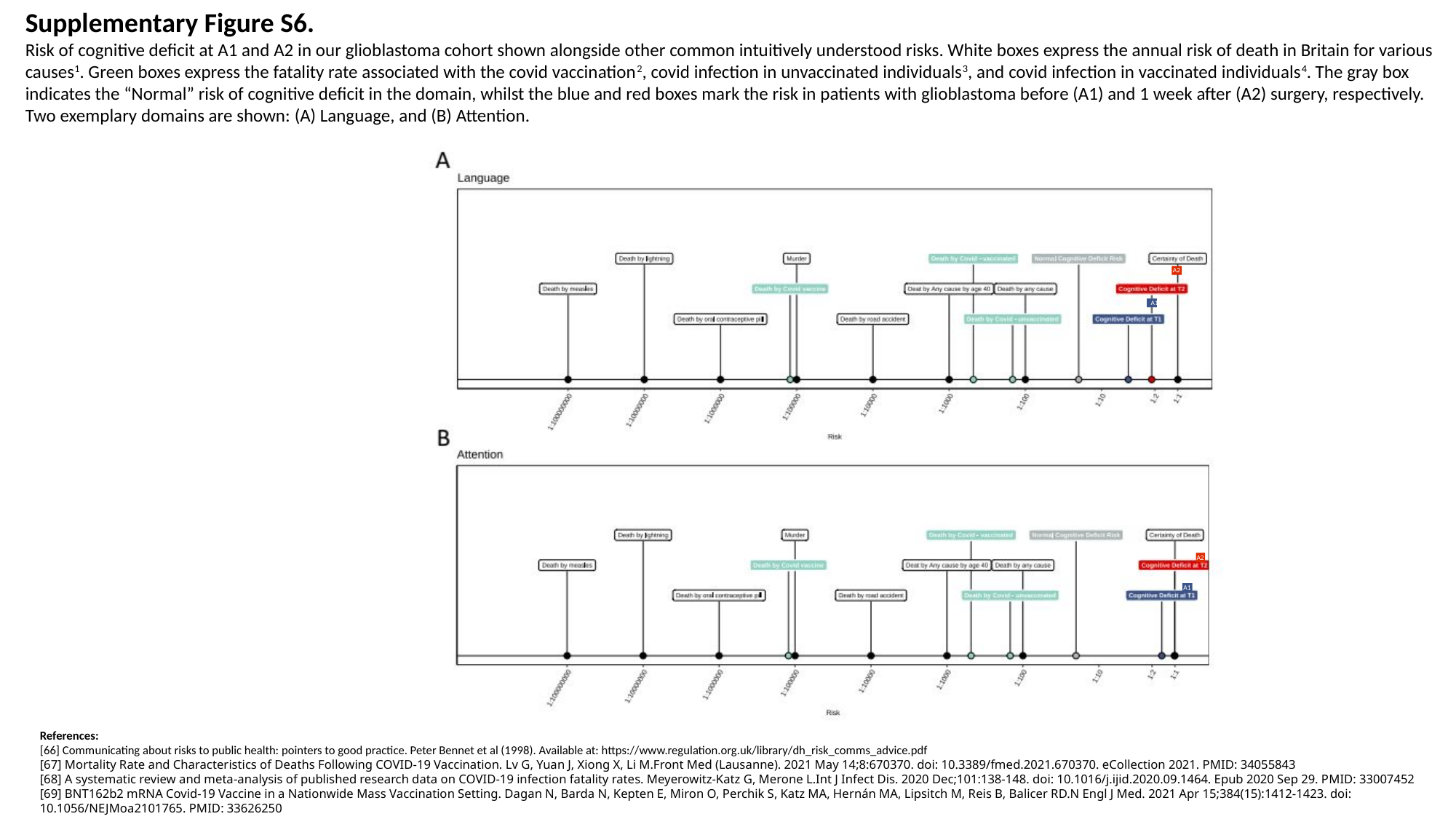

Supplementary Figure S6.
Risk of cognitive deficit at A1 and A2 in our glioblastoma cohort shown alongside other common intuitively understood risks. White boxes express the annual risk of death in Britain for various causes1. Green boxes express the fatality rate associated with the covid vaccination2, covid infection in unvaccinated individuals3, and covid infection in vaccinated individuals4. The gray box indicates the “Normal” risk of cognitive deficit in the domain, whilst the blue and red boxes mark the risk in patients with glioblastoma before (A1) and 1 week after (A2) surgery, respectively. Two exemplary domains are shown: (A) Language, and (B) Attention.
A2
A1
A2
A1
References:
[66] Communicating about risks to public health: pointers to good practice. Peter Bennet et al (1998). Available at: https://www.regulation.org.uk/library/dh_risk_comms_advice.pdf
[67] Mortality Rate and Characteristics of Deaths Following COVID-19 Vaccination. Lv G, Yuan J, Xiong X, Li M.Front Med (Lausanne). 2021 May 14;8:670370. doi: 10.3389/fmed.2021.670370. eCollection 2021. PMID: 34055843
[68] A systematic review and meta-analysis of published research data on COVID-19 infection fatality rates. Meyerowitz-Katz G, Merone L.Int J Infect Dis. 2020 Dec;101:138-148. doi: 10.1016/j.ijid.2020.09.1464. Epub 2020 Sep 29. PMID: 33007452
[69] BNT162b2 mRNA Covid-19 Vaccine in a Nationwide Mass Vaccination Setting. Dagan N, Barda N, Kepten E, Miron O, Perchik S, Katz MA, Hernán MA, Lipsitch M, Reis B, Balicer RD.N Engl J Med. 2021 Apr 15;384(15):1412-1423. doi: 10.1056/NEJMoa2101765. PMID: 33626250
